# *Escherichia coli* pathotypes and *Shigella* sero-groups in diarrheic children in Nairobi city, Kenya

**Published:** 2017

**Authors:** Peter Lokamar Nyanga, Jackson Onyuka, Mark Kilongosi Webale, Tom Were, Valentine Budambula

**Affiliations:** 1 *Disease Surveillance and Response Unit, Ministry of Health, Nairobi, Kenya.*; 2 *Department of Medical Laboratory Sciences, Mount Kenya University, Thika, Kenya.*; 3 *School of Health Sciences, Kirinyaga University, Kirinyaga, Kenya.*; 4 *Department of Medical Laboratory Sciences, Masinde Muliro University of Science and Technology, Kakamega, Kenya.*; 5 *Department of Environmental Health Sciences, Technical University of Mombasa, Mombasa, Kenya. *; 6 *Department of Medical Laboratory Sciences, Mount Kenya University, Thika, Kenya *

**Keywords:** E. coli pathotypes, Shigella sero-groups, Antimicrobial profile.

## Abstract

**Aim:**

In the present study, we investigated the prevalence of *E. coli* pathotypes and *Shigella* sero-groups and their antimicrobial profiles among diarrheic children in Nairobi city, Kenya.

**Background:**

Although diarrheagenic *E. coli* pathotypes and *Shigella* sero-groups are leading causes of diarrhea in children under five years in developing countries, their distribution and antimicrobial resistance vary from place to place and over time in a given region.

**Methods:**

In a cross-sectional study, we enrolled diarrheic children (n=354) under five years seeking treatment at Mbagathi Hospital, Nairobi city, Kenya,. Stool samples were collected from all children for bacterial culture. Bacterial isolation and identification was performed by conventional microbiological methods. Polymerase chain amplification was used to detect aspU, aggR, andpcvd432 for EAEC, est and elt for ETEC, eae for EPEC, stx for EHEC, and ipaH for EIEC and Shigella species. Antimicrobial profile was determined by disk diffusion method.

**Results:**

The prevalence of EAEC, ETEC, EPEC (eae), EIEC (ipaH) was 21.2%, 10.5%, 4.5%, and 0.6%, respectively, while that of mixed infection was 0.6%for ETEC/EAEC and 0.3%for EAEC/EPEC/ETEC. No EHEC strain was isolated. Pathogenetic analysis for EAEC showed that5.9% carried aspU,8.2% possessed both aspU and aggR and 7.1% had a combination of aspU, aggR andpcvd432 while that of ETEC was 2.3% for elt, 6.5% for both elt and est and 1.7% for est. The combination of aspU with aggR, elt and est, and pcvd432 with aggR, aspU and est was 0.3% for each case of ETEC/EAEC mixed infection. The aspU gene co-existed with aggR, pcvd432, eae and elt in the EAEC/EPEC/ETEC mixed infection. The prevalence of *S. boydii*, *S. dysenteriae*, *S. flexneriand,* *S. sonnei* was 0.8%, 0.6%, 1.7%, and 0.8%, respectively. No *E. coli* pathotype and *shigella* co-infection was detected. In addition, both *E. coli* pathotypes and *Shigella* species were resistant to ampicillin, trimethoprim/sulfamethoxazole, streptomycin, chloramphenicol and tetracycline while gentamycin and kanamycin resistance occurred in diarrheagenic *E. coli.*

**Conclusion:**

*E. coli* pathotypes and *Shigella* sero-groups harboring virulent genes are important causes of diarrhea in children in Kenya. The increasing spectrum of antibiotic resistance in diarrheagenic *E. coli* and *Shigella* species necessitates the development of antimicrobial stewardship education-programs to influence prescribing behavior as well as optimizing the use of effective antimicrobials in Kenya.

## Introduction

 Diarrheal diseases are the second leading cause of mortality in children under five years accounting for 1.7 billion episodes and more than 0·5 million deaths globally, most of which occur in developing countries (1). *Shigella* sero-groups and *Escherichia coli* pathotypes are important etiology of diarrhea mostly in children younger than 5 years in developing countries (2, 3). It is estimated that *Shigella* causes 164 million cases of bloody diarrhea and more than one million deaths while *E. coli* pathotypes accounts for about 56 million diarrhea episodes and more than 0.2 million deaths annually, the majority of which occur in children less than five years of age (2-5). 

There are six diarrheagenic *E. coli* (DEC) pathotypes which are differentiated from intestinal flora *E. coli* by the presence of virulence genes. This include *aafII*, *ast*, *aggR*, *aaspU and pcvd432 genes for *enteroaggregative *E. coli* (EAEC), *eae* and *bfp* genes for enteropathogenic *E. coli* (EPEC), *stx1* and *stx2 genes for* enterohemorrhagic *E. coli* (EHEC), heat stable (e*st*) and labile (e*lt)* toxin genes for enterotoxigenic *E. coli* (ETEC), *virf*, *ipaH* and *ipaL* genes for enteroinvasive *E. coli* (EIEC), and *daaE* gene for diffusely adherent *E. coli* (DAEC) *(*6*-*8*)*. Specific virulent genes for the seventh *E. coli* pathotype, crohn's disease-associated adherent-invasive *E. coli* (AIEC), have not been discovered to date. AIEC phenotypic identification is based on their ability to replicate within intestinal epithelial cells and macrophages (9, 10). In Kenya, conflicting reports on predominant diarrheagenic *E. coli* pathotype in adults and children (11-15) suggest that the prevalence of diarrheagenic *E. coli* pathotypes as etiology of diarrhea vary from region to region and even between adults and children in a country (16, 17). Previous study in Nairobi city, Kenya, identified EPEC as the major cause of diarrhea in children (15). However, this finding (15) is unreliable since one virulence gene was used to identify EAEC despite the heterogeneous nature of EAEC virulence factors (8, 18).

*Shigella* species is represented by four sero-groups namely *Shigella dysenteriae, S. flexneri, S. boydii *and* S. sonnei*. *Shigella *sero-groups are further classified based on structural difference of O-antigens polysaccharide into at least 15 serotypes of *S. dysenteriae,* 19 serotypes of* S. flexneri*, 20 serotypes of *S. boydii *and one serotype of *S. sonnei* (19) indicating the effectiveness of vaccine-induced serotype-specific protection (20). The severity of *shigellosis* vary within the sero-groups (21) and is linked to the expression of several virulence genes such as the invasion-associated locus (*ial*), the invasion plasmid antigen H (*ipaH*), *ipaBCD*, *Shigella* enterotoxin 1 and 2 (ShET-1 and ShET-2), *ipgD*, *icsA* and *virA* genes associated with colonization, invasion, intracellular survival and toxin-mediated disease (22). *IpaH* gene is carried by all four *Shigella* sero-groups and is used for the molecular diagnosis of shigellosis (22, 23). Variation in the prevalence of *Shigella *sero-groups among adults and children in Kenya (2, 24-27) suggest that the distribution of *Shigella* sero-group is heterogeneous over time and place in a country (21). To our knowledge, however, the distribution of *Shigella* sero-groups among diarrheic children under five years in Nairobi city, Kenya, has not been reported. 

Antimicrobial resistance in *Shigella* species and *E. coli* has been reported globally and increasing level of resistance is a growing concern (28). In Kenya, the vast majority of antibiotics prescriptions are made based on empirical diagnosis driving resistance among enteric pathogens (25) at this time when development of new antibiotics has gone down (29). Previous studies determined antimicrobial susceptibly patterns of *Shigella* and *E. coli *in Kenya (24, 25, 27, 30-32). Since antimicrobial resistance of *E. coli* and *Shigella* vary by region and over time (33), continuous antimicrobial surveillance is key in preventing emergence of antimicrobial resistant strains as well as guiding effective treatment (28). Therefore, this study determined diarrheagenic *E. coli* pathotypes and *Shigella* sero-groups and their antimicrobial susceptibility patterns in diarrheic children in Nairobi city, Kenya. 

## Methods

**Study site, design and population**

This cross-sectional study was conducted in Nairobi city, Kenya. Participants were diarrheic children under five years, seeking treatment at Mbagathi hospital, Nairobi city, Kenya. Diarrhea was defined, according World Health Organization (WHO) guidelines as the occurrence of three or more loose, liquid, or watery stools in a 24-hour period (34). A questionnaire was used to obtain information on age, gender, travel history, nausea, vomiting, abdominal pain, and diarrhea history of the children from the parents/guardians. Additional information on occupation of the guardian and water source and treatment was recorded on questionnaire. Clinical features such as body temperature, and dehydration signs were collected by clinicians. Stool specimens from diarrheic children were collected using wide mouthed, dry, leak-proof, sterile plastic containers and microbiology laboratory analysis performed within two hours of collection. Samples from children who had received antibiotics were excluded from the study.

**Bacteriological procedures**

Stool samples were plated on MacConckey agar (MCA), Xylose lysine deoxycholate agar (XLD), and Sorbitol MacConkey agar (SMAC) and incubated at 37°C overnight for the isolation and identification of *E.coli* and *Shigella* species. Identification of *E.coli* and *Shigella* species was performed by following the WHO recommendations (35). *Shigella* sero-goups were determined using serotyping kit (Denka Seiken Co. Ltd., Tokyo, Japan). The *E. coli* and *Shigella* isolates were further characterized for the presence of virulence genes.

**Detection of diarrheagenic** ***E. coli*** **pathotype and** ***Shigella*** **virulence genes**

**Figure 1:**
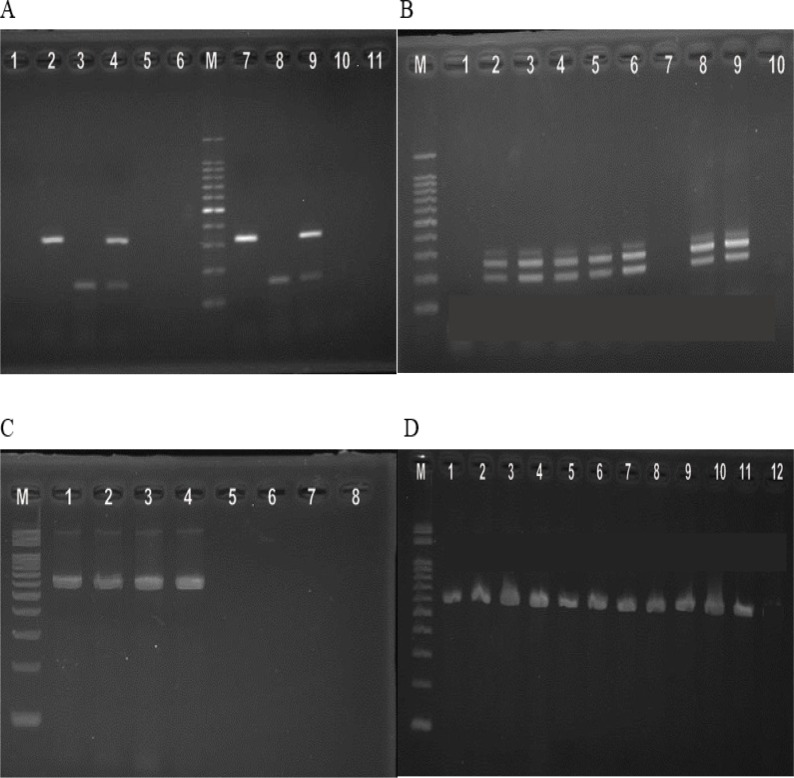
**A)** Multiplex PCR for detecting ETEC pathotype. Lane M: molecular weight marker. Lanes 1 and 11: Negative control. Lane 2 and 7: e*lt* (322 bp). Lanes 3 and 8: *est* (147 bp). Lanes 4 and 9: e*lt* (322 bp) and *est* (147 bp). Lane 5, 6 and 10: *elt* and *est*-gene negative *E. coli* isolates. **(B)**Multiplex PCR for detecting EAEC, EIEC, and EPEC pathotypes. Lanes 2,3,4,5,6,8 and 9: EAEC-*aggR* (254 bp) and *aspU* (282 bp). Lanes: 1 and 7: *aggR, ipaH, and aspU*-gene negative samples.Lane 10: Negative control. **(C)** Multiplex PCR for detecting EAEC, EIEC, and EPEC pathotypes. Lane M: Molecular weight marker. Lanes 1, 2, 3, 4:EIEC-*ipaH*-gene (600 bp). Lane 5,6 and 7: *ipaH*-gene negative sample. Lane 8: Negative control. **(D)** Singleplex PCR for detecting *shigellaipaH*-gene. Lane M: Molecular weight marker. Lanes 1,2,3,4,5,6,7,8, 9, 10 and 11: *ipaH*(619 bp). Lane 12: Negative control

DNA from cultured isolates of *E. coli* and *Shigella* species were extracted using QIAamp® DNA Mini Kit (QIAGEN GmbH, Hilden, Germany) according to the manufacturer’s recommendations. Initially, multiplex PCR to detect ETEC was performed using virulence gene-specific primers to detect *est* and *elt* for ETEC (Table 1) (6). This was followed by multiplex PCR with primers to detect *aspU, aggR, and pcvd432* for EAEC, *eae* for EPEC, stx for EHEC, and *ipaH for *EIEC (figure 1) (6). Singleplex PCR to detect *ipaH*-gene of *Shigella *sero-group isolates was performed using previously published primers (Table 1) (6). PCR reaction was carried out with 2.5 μL of the template DNA added to 47.5 μL mix containing, DreamTaq Green PCR Master Mix, nuclease free water and 1.0 μM of each primer (Thermo Fisher Scientific Inc., Waltham, Massachusetts, USA). Cycling conditions were initial denaturation at 95°C for 2 minutes, followed by 35 cycles of denaturation at 95°C for 30 seconds, annealing at 55°C for 30 seconds, extension at 72°C for 1 minute and final extension at 72°C for 10 minutes. The amplified DNA products were visualized by agarose gel electrophoresis method.

**Table 1:** PCR primers used in this study

Primers, Genes and Sequence (5'- 3')	Amplicon size (bp)	Target gene
SK1 CCCGAATTCGGCACAAGCATAAGC SK2 CCCGGATCCGTCTCGCCAGTATTCG	881	*eae*
VTcom-u: GAGCGAAATAATTTATTATGTG VTcom-d: TGATGATGGCAATTCAGTAT	518	*stx1* and *stx2*
AL65: TTAATAGCACCCGGTACAAGCAGG AL125: CCTGACTCTTCAAAAGAGAAAATTAC	147	*est*
LT1: TCTCTATGTGCATACGGAGC LTr: CCATACTGATTGCCCGCAAT	322	*eltB*
ipaIII: GTTCCCTTGACCGCCTTTCCGATACCGTC ipaIV: GCCGGTCAGCCACCCTCTGAGAGTAC	600	*IpaH*
aggRks1: GTATACACAAAAGAAGGAAGC aggRks2: ACAGAATCGTCAGCATCAGC	254	*aggR*
Eaggfp: AGACTCTGGCGAAAGACTGTATC Eaggbp: ATGGCTGTCTGTAATAGATGAGAAC	194	*Pcvd432*
aspU-3: GCCTTTGCGGGTGGTAGCGG aspU-2: AACCCATTCGGTTAGAGCAC	282	*aspU*

**Antimicrobial susceptibility profile**

The bacterial isolates were tested for antibiotic resistance by the disk diffusion method according to established standard operating procedures (36). The antibiotics tested included ampicillin, trimethoprim/sulfamethoxazole, ceftriaxone, streptomycin, amoxicillin/clavulanic acid, gentamycin, kanamycin, ciprofloxacin, chloramphenicol, erythromycin, nalidixic acid and tetracycline.

**Data analysis**

Statistical analyses were performed using SPSS version 19.0 for Windows (IBM SPSS Statistics for Windows, Version 19.0. Armonk, NY: IBM Corp.). Descriptive statistics were used to analyze the data and the results expressed as frequency and percentage. 

**Ethical considerations**

This study was ethically approved by Kenyatta National Hospital/University of Nairobi (KNH-UoN) Ethics and Research Committee and was conducted according to Helsinki declarations. A consent form was read and signed by either parent or guardian of each child. Diarrheic children were treated by clinicians according to World Health Organization guidelines for treatment of diarrhea in children (34). All study participants' information and test results were confidentially kept.

## Results

**Demographic and clinical information of study participants**

The demographic and clinical information of the diarrheic children under five years of age in Nairobi, Kenya, is presented in table 2. A total of 354 children with diarrhea were included in the study. The age distribution showed that 242 (68.4%) children were within the age group 1 and 30 months and 112 (31.6%) children were between 31 and 60 months. The overall gender distribution was 170 (48%) female and 184 (52%) male. Guardians of 351 (99.2%) and 3 (0.8%) children reported using piped and borehole water, respectively. In addition, guardians of 206 (58.6%) and 10 (2.8%) children, respectively, reported treating drinking and having travelled to their rural homes two weeks prior to the start of the study. Occupation distribution showed that two (0.6%), 17 (4.8%), 5 (1.4%), 14 (4.0%), and 178 (50.3%) of the guardians were in the health care practitioner, office administrative support, construction/installation/repair, education/training, and sales, respectively, while 138 (39.0%) were unemployed. 

Temperature of <38.0°C and ≥ 38.0°C was recorded in 54 (15.3%) and 300 (84.7%) children, respectively. In this study, 293 (82.8%), 32 (9.0%) and 29 (8.2%), respectively, reported having diarrhea for 1-3, 4-6 and ≥7 days. Of which, 299 (84.5%) were mucoid diarrhea and 55 (15.5%) were bloody diarrhea. Vomiting was evidenced in 280 (79.1%) patients, fever in 293 (82.8%), abdominal cramp in 235 (66.4%), headache in 11 (3.1%), nausea in 48 (13.6%), and appetite loss in 326 (92.1%) children. Clinical diagnosis of dehydration revealed that 295 (83.3%) had sunken eyeballs, 110 (31.1%) children had dry tongue and 182 (51.4%) had reduced skin elasticity.

**Prevalence of diarrheagenic** ***E. coli*** **pathotypes and** ***Shigella*** **sero-groups isolated from study participants**

The prevalence of diarrheagenic *E. coli* pathotypes and *Shigella* sero-groups in diarrheic children under five years of age in Nairobi City, Kenya, is presented in table 3. The prevalence of EAEC, ETEC, EPEC (*eae*), EIEC (*ipaH*), was 75 (21.2%), 37 (10.5%), 16 (4.5%), and 2 (0.6%) pure strains, respectively, while the prevalence of mixed infections was 2 (0.6) for ETEC/EAEC and 1 (0.3) for EAEC/EPEC/ETEC. No EHEC strain was isolated from the children. Among the EAEC pure isolate infections, 21 (5.9%) had *aspU *gene, 29 (8.2%) had both *aspU *and* aggR *genes and 25 (7.1%) had a combination of *aspU, aggR *and* pcvd432* genes among the EAEC pure isolate infections. The pathogenetic profile of ETEC pure isolate infections was 8 (2.3%) for *elt* gene, 23 (6.5%) for both *elt *and* est *genes and 6 (1.7%) for *est* gene. The combination of *aspU *with* aggR, elt *and* est, *and *pcvd432 *with* aggR, aspU *and* est* was detected in 1(0.3%) case each in ETEC/EAEC mixed infection. The *aspU* gene co-existed with *aggR, pcvd432, eae *and* elt* in the 1 (0.3%) EAEC/EPEC/ETEC mixed infection case.

**Table 2:** Demographic and clinical information of study participants

Characteristics	Number (%)
Age in months	
1-30	242 (68.4)
31-60	112 (31.6)
Gender	
Female	170 (48)
Male	184 (52)
Source of water	
Piped water	351 (99.2)
Borehole	3 (0.8)
Water treatment	206 (58.2)
Travel history	10 (2.8)
Occupation of guardian	
Health care practitioner	2 (0.6)
Office/administrative/support	17 (4.8)
Construction/installation/repair	5 (1.4)
Education/ training	14 (4.0)
Sales	178 (50.3)
Unemployed	138 (39.0)
Body temperature	
<38.0	54 (15.3)
≥ 38.0	300 (84.7)
Duration of diarrhea	
1-3	293 (82.8)
4-6	32 (9.0)
≥7	29 (8.2)
Diarrhea type	
Mucoid	299 (84.5)
Bloody	55 (15.5)
Symptoms	
Vomiting	280 (79.1)
Fever	293 (82.8)
Abdominal cramp	235 (66.4)
Headache	11 (3.1)
Nausea	48 (13.6)
Appetite loss	326 (92.1)
Sunken eyeball	295 (83.3)
Dry tongue	110 (31.1)
Reduced skin elasticity	182 (51.4)

Data are presented as number and proportions (%) of study participants. ≤, less than or equal to. <, less than. ≥, greater than or equal to. >, greater than. Health care practitioner (Nurse, Clinical officer). Office administrative support (secretary, clerical officer, social worker, driver, househelp, and caretaker). Construction/installation/repair (welder, carpenter, mason, tailor). Education/ training (teacher).Sales (saloonist, hawkers and small scale business, sales agents

A total of 14 (4.0%) *Shigella* isolates were observed in this study comprising of 3 (0.8%) *S. boydii*, 2 (0.6%) *S. dysenteriae*, 6 (1.7%) *S. flexneri* and 3 (0.8%) *S. sonnei*. All the *Shigella* isolates harbored *ipaH* gene. No *shigella* and pathogenic *E. coli* co-infection was detected in this study.

**Antimicrobial susceptibility patterns of diarrheagenic** ***E. coli*** **and** ***Shigella species***

The antimicrobial susceptibility patterns of the diarrheagenic *E. coli* and *Shigella* species is presented in **table 4**. About 77.4%, 66.8%, 3.0%, 80.5%, 14.3%, 72.2%, 56.4%, 9.0%, 75.9%, 1.5%, 11.3%, 64.7% of DEC was resistant to ampicillin, trimethoprim/sulfamethoxazole, ceftriaxone, streptomycin, amoxicillin/clavulanic acid, gentamycin, kanamycin, ciprofloxacin, chloramphenicol, erythromycin, nalidixic acid, and tetracycline, respectively. Although none of the *Shigella* isolate was resistant to gentamycin, kanamycin and erythromycin, 85.8%, 57.1%, 14.3%, 92.9%, 7.1%, 14.3%, 57.1%, 14.3%, 85.8%, of the children were infected with *Shigella *species resistant to ampicillin, trimethoprim/ sulfamethoxazole, ceftriaxone, streptomycin, amoxicillin/ clavulanic acid, ciprofloxacin, chloramphenicol, nalidixic acid, and tetracycline, respectively.

## Discussion

The prevalence diarrheagenic *E. coli* pathotypes and *Shigella* sero-groups as etiologic agents of diarrhea vary considerably from region to region and over time in a given region (17, 21, 37, 38). In addition, clinical manifestations of *E. coli* and *shigella* species are influenced by the type of virulence factor present which differ by pathotype and sero-group, respectively (8, 21). While antibiotics have proved successful in the treatment of *E. coli* and *Shigella* infections, emergence and spread of acquired and transmitted antimicrobial resistant strains is common (33). Thus, continuous epidemiological surveillance is key for planning antimicrobial treatment.

The findings of this study showing the presence of *aspU, aggR, pcvd432, eae, elt, est, *and *ipaH*, virulent factors in *E. coli* pathotypes, partly mirrors previous studies in Nairobi city, Kenya (14, 15). The EAEC predominates in this study with the remainder being EIEC, EPEC, ETEC and mixed diarrheagenic *E. coli* infections is partly consistent with previous studies in Kenya (14). This observation may be attributed to zinc deficiency that affects about 61% of children under five years in Kenya (39). Zinc inhibits diarrheagenic *E. coli* endothelial adherence, biofilm formation, virulence gene expression as well as promoting host immune clearance of diarrheagenic *E. coli* (40, 41). This and other studies in Kenya did not detect EHEC (15). Perhaps, EHEC is predominantly present in the environment and reservoirs, and it does not play role in infantile diarrhea (8, 42). However, the findings of this study are inconsistent with previous studies that identified EPEC as the most common DEC in children under five years in Nairobi city, Kenya (15). Probably, because of the proved EAEC genome heterogeneity (8, 18), using one virulence gene decreased the rate of isolation in that study (15). Taken together, EAEC, EIEC, EPEC and ETEC pathotypes harboring virulent factors are an important etiology of diarrhea in Kenyan children and require more attention from our public health services.

**Table 3.** Prevalence of *E. coli* pathotype and *Shigella* sero-groups

Isolate type	Strain	Number (%)
Diarrheagenic* E. coli* pathotypes		
EAEC (all)		75 (21.2)
	*aspU*	21 (5.9)
	*aspU/aggR*	29 (8.2)
	*aspU/aggR/pcvd432*	25 (7.1)
EHEC	*Stx*	0 (0.0)
EPEC	*Eae*	17 (4.8)
ETEC (all)		37(10.5)
	*Elt*	8 (2.3)
	*elt/est*	23 (6.5)
	*Est*	6 (1.7)
EIEC	*ipaH*	5 (1.4)
ETEC/EAEC (all)		2 (0.6)
	*aspU/aggR/elt/est*	1 (0.3)
	*aspU/aggR/pcvd432/est*	1 (0.3)
EAEC/EPEC/ETEC	*aspU/aggR/pcvd432/eae/elt*	1 (0.3)
*Shigella* sero-groups		
*S. boydii*	*ipaH*	3 (0.8)
*S. dysenteriae *	*ipaH*	2 (0.6)
*S. flexneri*	*ipaH*	6 (1.7)
*S. sonnei*	*ipaH*	3 (0.8)
		

Data are presented as number and proportions (%) of isolates. EPEC, enteropathogenic E. coli. ETEC, enterotoxigenic E. coli. EAEC, enteroaggregative E. coli. EIEC, enteroinvasive E. coli. EHEC.*Shigella boydii.S. dysenteriae*, *Shigella dysenterae.S. flexneri,Shigella flexneri*. *S. sonnei, Shigella sonnei*

Several copies of *ipaH* gene being present on both plasmids and chromosomes may explain the gene being tested positive in all *Shigella* sero-groups (43) in this and previous studies (22, 23). The findings of this study reporting *Shigella flexneri* as the commonest strain and the remainder being *S. dysenteriae, S. boydii* and *S. sonnei*, although in low rates, is partly consistent with previous studies in Kenya (25-27). *Shigella flexneri* is less virulent than other* Shigella* sero-groups because it doesn’t kill its host immediately (44) explaining the dominancy of *S. flexneri* in Nairobi city, Kenya. However, the findings of this study are inconsistent with previous studies in industrialized countries reporting the dominancy of *S. sonnei* (45). Industrialized countries have greater levels of wealth and economic development positively influencing hospital care and treatment for diarrhea and dysentery (37, 46) which may drive the dominancy of *S. sonnei* due to its greater ability to develop resistance to antibiotic treatment (47). Therefore, heterogeneous distribution of *Shigella *species suggests that multivalent vaccine will be needed to prevent shigellosis in children in Kenya.

Many of the diarrheagenic *Escherichia coli* were resistant to ampicillin, chloramphenicol, tetracycline, trimethoprim/sulfamethoxazole, gentamycin, kanamycin, and streptomycin, which is partly consistent with the findings of previous studies in Kenya (24, 30, 31). On the other hand, increasing susceptibility of diarrheagenic *E. coli* to ampicillin, chloramphenicol, gentamycin, tetracycline, and trimethoprim/sulfamethoxazole has been reported among the Maasai community of Kenya (32). This is possibly due to the lower levels of exposure and usage of antimicrobials among the Maasai community of Kenya who mostly practice traditional medicine (48). The findings of this study showing *Shigella* species resistance to Ampicillin, trimethoprim/ sulfamethoxazole, streptomycin, chloramphenicol and tetracycline are in agreement with previous studies in Kenya (27) and Ethiopia (49). Likewise, the susceptible of *Shigella* species towards, ceftriaxone, amoxicillin/clavulanic acid, gentamycin, kanamycin, ciprofloxacin, erythromycin, and nalidixic acid is congruent to previous studies in Ethiopia (50). However, *Shigella* resistance to ceftriaxone, amoxicillin/clavulanic acid, gentamycin, kanamycin, ciprofloxacin, erythromycin, and nalidixic acid have been reported in China (51) and Iran (52) highlighting the importance of judicious use of these drugs to preserve their effectiveness for treatment in Kenya. Taken together, antimicrobial stewardship education-programs have to be developed to influence prescribing behavior hence optimizing the use of effective antimicrobials in Kenya. 

It is important to outline the limitations of this study. The findings of this study must be interpreted with caution because molecular diagnosis of *E. coli* and *Shigella* species was performed on cultures but not stool samples. The presence of DAEC and AIEC pathotypes were not investigated in this study. In this study, the primers to detect the bundle-forming pilus (*bfpA*) gene which is present in typical EPEC and absent in some atypical EPEC were not included in diarrheagenic *E. coli* pathotyping since further analysis of *eae*-positive isolates for the presence of *stx*-gene is sufficient to distinguish EPEC from EHEC (6, 53). Although *Shigella* species expresses several virulent factors (22), singleplex PCR targeting *ipaH* gene was used in this study. We acknowledge the small sample size of *Shigella* isolates assayed for antimicrobial sensitivity.

We conclude that diarrheagenic *E. coli* pathotypes and *Shigella *sero-groups are an important etiology of diarrhea in children under five years in Kenya. These pathogens are of public health importance since they are highly heterogeneous and harbor very threatening virulent genes like *aspU, aggR, pcvd342*, *est*, *elt*, *eae*, *stx*, and *ipaH *for diarrheagenic* E. coli *pathotypes and* ipaH *for *Shigella *sero-groups. The heterogeneity of* Shigella *sero-groups is important in the implementation of vaccine prevention strategies. In addition, both diarrheagenic* E. coli *and* Shigella *species are resistant to ampicillin, trimethoprim/sulfamethoxazole, streptomycin, chloramphenicol and tetracycline while gentamycin and kanamycin resistance occur in diarrheagenic *E. coli. *This result is important in the treatment and prevention of the spread of antimicrobial resistant diarrheagenic *E. coli* pathotype and *shigella* species.
